# Human perceptual learning is delayed by the N-methyl-D-aspartate receptor partial agonist D-cycloserine

**DOI:** 10.1177/0269881120986349

**Published:** 2021-02-11

**Authors:** Harriet Dempsey-Jones, Susann Steudte-Schmiedgen, Michael Browning, Tamar R Makin, Marcella L Woud, Catherine J Harmer, Juergen Margraf, Andrea Reinecke

**Affiliations:** 1 Wellcome Centre for Integrated Neuroimaging, University of Oxford, Oxford, UK; 2 School of Psychology, University of Queensland, Brisbane, Australia; 3 Department of Psychotherapy and Psychosomatic Medicine, Technische Universität Dresden, Dresden, Germany; 4 Department of Psychiatry, University of Oxford, Oxford, UK; 5 Oxford Health NHS Foundation Trust, Oxford, UK; 6 Institute of Cognitive Neuroscience, University College London, London, UK; 7 Department of Clinical Psychology and Psychotherapy, Ruhr-University Bochum, Bochum, Germany

**Keywords:** N-methyl-D-aspartate, somatosensory plasticity, touch perception, psychophysics, cognitive enhancement

## Abstract

**Background:**

The optimisation of learning has long been a focus of scientific research, particularly in relation to improving psychological treatment and recovery of brain function. Previously, partial N-methyl-D-aspartate agonists have been shown to augment reward learning, procedural learning and psychological therapy, but many studies also report no impact of these compounds on the same processes.

**Aims:**

Here we investigate whether administration of an N-methyl-D-aspartate partial agonist (D-cycloserine) modulates a previously unexplored process – tactile perceptual learning. Further, we use a longitudinal design to investigate whether N-methyl-D-aspartate-related learning effects vary with time, thereby providing a potentially simple explanation for apparent mixed effects in previous research.

**Methods:**

Thirty-four volunteers were randomised to receive one dose of 250 mg D-cycloserine or placebo 2 h before tactile sensitivity training. Tactile perception was measured using psychophysical methods before and after training, and 24/48 h later.

**Results:**

The placebo group showed immediate within-day tactile perception gains, but no further improvements between-days. In contrast, tactile perception remained at baseline on day one in the D-cycloserine group (no within-day learning), but showed significant overnight gains on day two. Both groups were equivalent in tactile perception by the final testing – indicating N-methyl-D-aspartate effects changed the timing, but not the overall amount of tactile learning.

**Conclusions:**

In sum, we provide first evidence for modulation of perceptual learning by administration of a partial N-methyl-D-aspartate agonist. Resolving how the effects of such compounds become apparent over time will assist the optimisation of testing schedules, and may help resolve discrepancies across the learning and cognition domains.

## Introduction

The scientific community has long been fascinated by the possibility of improving our natural human capacities through intervention or technology. One avenue to promote learning in humans and non-humans has been by administration of N-methyl-D-aspartate (NMDA) agonist drugs, such as D-cycloserine (Kalisch et al., 2009; Onur et al., 2010; Reinecke et al., 2020; Volianskis et al., 2015). D-cycloserine is an antibiotic, originally developed for the treatment of tuberculosis, that is a high-affinity partial agonist for NMDA receptors. Aligned with the surge of interest in other so-called ‘cognitive enhancement’ drugs (Cakic, 2009; Greely et al., 2008), D-cycloserine has been repurposed from its original medical application in attempts to improve cognitive functions and learning.

Supporting its efficacy in this role, some studies report that acute doses of D-cycloserine can enhance procedural and declarative learning, fear learning and extinction, and place learning – supposedly by triggering processes of neural plasticity (in humans: Feld et al., 2013; Forsyth et al., 2015; Kuriyama et al., 2011; Onur et al., 2010; in rodents: Golden and Houpt, 2007; Monahan et al., 1989; Walker et al., 2002). The processes modulated by D-cycloserine suggest that it may be particularly effective in altering synaptic plasticity in amygdala (Britton et al., 2007; LeDoux, 2000) and hippocampal brain circuits (Onur et al., 2010).

In the current study, we ask whether administration of an NMDA receptor agonist compound modulates a previously unexplored learning process: tactile perceptual learning – the enhancement of touch perception by training (Dempsey-Jones et al., 2016, 2019; Harrar et al., 2014; Harris et al., 2001). D-cycloserine has a modulatory effect on decision-making areas, as demonstrated by changes in neural activity (Aupperle et al., 2009) and in behaviour (Scholl et al., 2014). Given this, investigation of whether D-cycloserine alters tactile learning could inform the on-going debate regarding whether this form of learning is driven primarily by changes in primary sensory areas (Recanzone et al., 1992) or by tuning read-out of sensory areas by higher-order, decision-making brain areas (Law and Gold, 2008; Shibata et al., 2011). If D-cycloserine were found to benefit tactile sensory learning – as evidenced variously in other learning domains (see above) – this could have important applications for boosting sensory training to rehabilitate degraded perception (e.g. after stroke), or to optimise normal human perception.

Further, we wished to investigate a potential explanation for the large body of research reporting null effects of D-cycloserine on various forms of learning (e.g. procedural learning: Cherry et al., 2014; Feld et al., 2013; Gunthner et al., 2016; reward learning: Scholl et al., 2014; declarative memory: Kuriyama et al., 2011; working memory: Forsyth et al., 2015; fear extinction: Guastella et al., 2007; extinction therapies: Kamboj et al., 2011; see Hofmann et al., 2013, 2015, for review). We suggest that one reason for the apparent mixed success of D-cycloserine may be that D-cycloserine has differential effects on learning and cognition over time. Here, tactile learning makes a good model for studying timing effects, as it has been documented to evolve over days (Dempsey-Jones et al., 2016).

Evidence for such drug timing effects comes from previous research indicating D-cycloserine has a greater effect on motor performance on the day subsequent to learning and drug administration, as compared to the day of learning/administration (i.e. during consolidation of motor learning; Kuriyama et al., 2011; also see Feld et al., 2013 for similar results in declarative memory). Additionally, previous work from our group suggests the possibility of an as yet undocumented detriment of the drug during its active phase – reflected in an increase in reaction times during motor learning (Gunthner et al., 2016). Such a detriment could be linked with antagonistic effects of D-cycloserine that occur when endogenous NMDA levels are high (Watson et al., 1990). Here, we therefore looked at whether NMDA-related learning effects might be different for online learning concurrent to perceptual training, versus consolidation of learning that occurs over an extended post-learning time-period (touch: Kaas et al., 2013; vision: Karni and Sagi, 1993).

In sum, we investigated whether D-cycloserine affects the process of perceptual learning, and whether these effects varied over time-course of learning. To this end, we trained participants to improve touch perception on their middle finger, testing tactile acuity at baseline, directly after training (within-day learning), and the following day (between-day learning). Half the participants were administered D-cycloserine before training, and the other half received a placebo. Psychophysical measures were extracted from testing data to track touch perception over time (tactile ‘thresholds’). We also extracted other psychophysical information regarding stimulus sensitivity and noise in the data (‘slope’ and ‘goodness of fit’ measures respectively, see Methods) to provide additional mechanistic insight regarding the influence of the drug.

We predicted a significant difference in tactile learning over time between the two groups. Specifically, in the D-cycloserine group we predicted that we would either see an attenuation of online learning while the drug was active (consistent with previous null results of D-cycloserine on learning, see above) or an interference in tactile learning while the drug was active (e.g. Gunthner et al., 2016, also see above) – as compared to the placebo controls. That is, a delay or negative impact on learning progression, respectively. Finally, we predicted that we may also see an enhancement of learning consolidation (Feld et al., 2013; Kuriyama et al., 2011) in the D-cycloserine group as compared to placebo. Overall, we anticipated an initial null effect (or decrement) caused by D-cycloserine, followed by enhanced consolidation would result in a slight enhancement of tactile thresholds for the drug compared to the placebo group by final testing.

## Methods

### Participants

Thirty-four individuals participated in the study from 40 recruited (three were removed due to drop out/equipment failure, three due to sensory thresholds at chance; see Table 1 for demographics). There were 16 in the D-cycloserine group (age, mean (M) = 22.44, 10 females, one left-handed), and 18 in the placebo group (age, M=24.06, eight females, one left-handed), randomly assigned to groups. Participants provided written informed consent and were reimbursed for their time. Please see Supplementary Material for a full list of exclusion criteria. Ethical study approval was granted by the Oxford Central University Research Ethics Committee. Please note: the 34 participants included in the final dataset also performed some tasks on an unrelated paradigm looking at emotional-bias learning and D-cycloserine that will not be discussed here (Woud et al., 2018).

**Table 1.** Socioeconomic, personality and psychological symptom questionnaire scores in the two groups (mean (M)±standard deviation (SD), independent-samples *t*-test/X^2^-test *p* scores). No differences were found between groups on any measure.

	D-cycloserine (*n*=16)	Placebo (*n*=18)	*p* Score
Age	23.8±4.6	22.4±3.6	0.33
Gender	8 female/8 male	8 female/10 male	0.57
Years of education	16.5±3.6	16.6±2.8	0.94
Verbal IQ (NART)	115.7±5.3	118.8±4.7	0.10
BMI (kg/m^2^)	22.9±3.6	22.2±2.1	0.51
Self-report questionnaires			
Trait anxiety (STAIT)	32.0±6.9	33.2±6.9	0.62
Neuroticism (EPQ-N)	6.1±4.3	8.2±3.8	0.15
Beck Depression Inventory	1.8±1.8	3.0±4.3	0.28
Behavioral activation (BAS)	25.5±4.7	24.4±5.8	0.57
Behavioral inhibition (BIS)	15.9±3.8	16.4±3.3	0.68
Attentional control - focusing (ACS)	26.5±3.5	25.2±3.5	0.27
Attentional control - shifting (ACS)	32.0±5.4	33.8±4.1	0.28

ACS: Attentional Control Scale; BAS: Behavioral Activation Scale; BIS: Behavioral Inhibition Scale; BMI: body mass index; EPQ: Eysenck Personality Questionnaire; IQ: intelligence quotient; NART: National Adult Reading Test; STAIT: Spielberger State Trait Anxiety Inventory.

### General experimental timeline and details

The experiment was a double-blind, placebo-controlled design. All groups participated in one block of tactile perception training on day 1. To assess changes resulting from training, there were three blocks of tactile perception testing – before, directly after and 24 h subsequent to training (though note: two participants from each group were tested 48 h later due to scheduling constraints). Participants fasted 2 h before their visit. On day 1, all participants underwent baseline testing, then received a single dose of D-cycloserine (King Pharmaceuticals) or a matching placebo capsule, microcrystalline cellulose 1:1 ratio (Klumpers et al., 2012; Onur et al., 2010). Previous studies using D-cycloserine in healthy volunteers have indicated a drug effect on single-session hippocampal learning with a 250 mg dose, but not with a 50 mg dose (Onur et al., 2010). Other work looking at therapeutic learning during cognitive-behaviour therapy with a D-cycloserine adjunct showed no differences between 50 mg and 500 mg on intervention outcomes (Ressler et al., 2004). Given this, a 250 mg dose was selected for use here. The drug has been shown to reach plasma peak levels within 1–4 h, and to have a half-life of 8–15 h (Patel et al., 2011; van Berckel et al., 1997, 1998). Thus, dosage levels allowed close-to-peak drug effects during training.

Two hours after drug/placebo administration, participants underwent ~30 min of training, and then a final testing (the online test) directly afterwards. The final test (consolidation test) was on day 2 (24/48 h later, see above). All testing blocks lasted ~20 min. Finally, we assessed for awareness of the intervention (‘Do you think you were in the drug or placebo condition?’) and side effects. There were no differences between groups on either measure (0.179 < *p* > 1.00).

During training and testing participants were blindfolded. They were instructed to prioritise task accuracy over speed, and no time limit was imposed. Stimuli presentation was controlled by a computer (MATLAB, release 2013a, MathWorks, Inc., Boston, Massachusetts, USA) and was pseudo-random (see below). Participants responded with a mouse (held in the hand not currently being used for testing/training).

### Stimuli for testing and training

**Figure 1.**
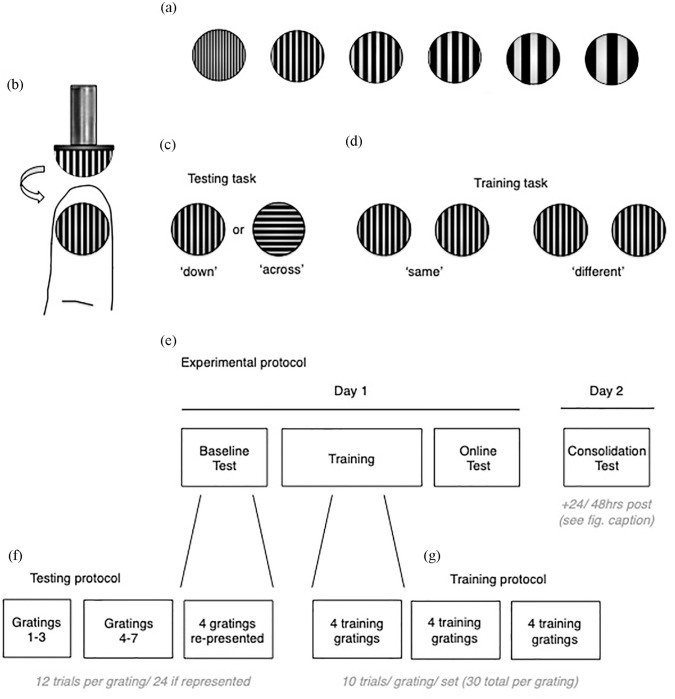
Grating stimuli used for testing and training. (a) Examples of six gratings varying in ridge/groove width (isometric). This allowed manipulation of task difficulty (smallest ridges/grooves were hardest to perceptually discriminate in orientation, largest were easiest to discriminate). (b) Gratings were manually applied to the distal fingertip. Example shows the ‘down’ orientation, i.e. the ridges/grooves were oriented down with respect to the proximo-distal axis of the finger. (c) and (d) Visual representation of the testing and training tasks, respectively. (e) Timeline of the experimental protocol. Short breaks were taken between each testing/training block. Please note that participants underwent the consolidation test 24 h after the baseline test, though two participants from either group did this test 48 h later, due to scheduling constraints. (f) Order and distribution of grating sets for testing and training. Short breaks were taken between each set.

A set of 11 tactile ‘grating’ stimuli were used for testing and training (JVP Domes, Stoelting, Wood Dale, Illinois, USA). Tactile gratings are commonly used in testing the spatial resolution of touch discrimination ability (Craig and Johnson, 2000; Dempsey-Jones et al., 2016, 2019; Harrar et al., 2014; Sathian and Zangaladze, 1996, 1997; Van Boven and Johnson, 1994; Vega-Bermudez and Johnson, 2001; Werhahn et al., 2002; Wong et al., 2011, 2013), and are the tactile equivalent of Gabor patches used in visual spatial discrimination testing (Lu et al., 2005). Each grating consists of a small plastic dome with grooves cut into the curved surface (see Figure 1), forming ridges that are of isometric width to the groove width.

Gratings were presented manually to the glaborous surface of distal finger pad. The finger remained static at all times. The participants’ task was to discriminate the orientation of the stimulus grooves (/ridges) with respect to their finger (precise task varied for testing/training, more below). Percentage accuracy was assessed and used to estimate the participant’s psychophysical threshold at each test (see Supplementary Material for details). Two orientations were presented, where the grooves were oriented either parallel or perpendicular to the proximo-distal axis of the finger. These orientations were described as ‘down’ and ‘across’, respectively. Please note: these orientations do not align with gravitational ‘up’/‘down’. Participants were trained in identification of these orientations, and the accompanying verbal labels in a short familiarisation (see Supplementary Material). The width of the ridges allowed the manipulation of perceptual discrimination difficulty – with larger ridges being easier to perceptually discriminate and smaller ridges being harder, i.e., coarse versus fine textures. The ridge sizes used were: 0.25, 0.5, 0.75, 1.0, 1.2, 1.5, 2.0, 2.5, 3.0 and 3.5 mm ridge/groove spacing. Each presentation lasted ~1 s, and there was ~2–3 s between stimulus presentations during the tasks (Bleyenheuft and Thonnard, 2007; Van Boven and Johnson, 1994).

### Testing task

Testing and training tasks were incorporated that have been extensively validated (Dempsey-Jones et al., 2016, 2019; Harrar et al., 2014). Participants underwent a brief familiarisation block before the first testing block began to introduce the stimuli/task, thereby improving the reliability of testing data (see Supplementary Material). The testing task was a simple orientation discrimination task, performed with the middle finger (left/right middle finger tested in interleaved sets, order randomised). On each testing trial, the experimenter would present one grating to the participant’s fingertip. Participants responded using a two-alternative forced choice (2AFC) whether the dome was in the down or across orientation (see above). Seven grating sizes were used for testing (0.75, 1.0, 1.2, 1.5, 2.0, 2.5 and 3.0 mm ridge/groove spacing). One grating size was selected for presentation per block (12 trials/set). Each grating was presented for a set, with order randomised, i.e. method of constant stimuli (Klein, 2001). Following this, four gratings were selected to be re-presented for an additional block each (see Figure 1). The grating sizes selected for additional testing were chosen based on the participant’s performance on that test up to that point. Specifically, they were chosen to be within the range where performance was variable (grating sizes with consistently high accuracy (+90%) were not repeated, to provide more performance information within the dynamic range). This protocol resulted in 132 total trials. No performance information was provided on accuracy trial-by-trial (to reduce learning from the testing protocol). General feedback on accuracy was provided, however, to improve task engagement over headphones after ¼ to ⅓ of the blocks (e.g. ‘100% correct’), timing randomised. Short breaks were taken after the fourth and seventh sets. During this time, participants were encouraged to take their blindfold off and rest for 1–2 min.

### No differences between fingers tested

Tactile sensory learning is known to spread from the trained finger to the homologous finger of the other hand (Dempsey-Jones et al., 2016, 2019; Harrar et al., 2014; Harris et al., 2001; Kaas et al., 2013; Nagarajan et al., 1998; Sathian and Zangaladze, 1998; Spengler et al., 1997), presumably due to overlap in representation of these fingers in the somatosensory system (Wesselink et al., 2019). We tested the trained finger and its homologous counterpart to see if D-cycloserine administration altered the transfer of learning from the trained to untrained finger.

We found D-cycloserine did not affect learning transfer. This was reflected in non-significant differences in tactile thresholds between the trained and homologous fingers across testing blocks, i.e. no difference in learning effects (0.196<*p* > 0.593; please see Supplementary Material for full analysis). Given the non-significant difference between fingers, in the interests of parsimony, we collapsed data for the two fingers for the remainder of our comparisons (e.g. threshold_average_=(threshold_trained_ + threshold_homologous_)/2). Note, however, that the picture of results was consistent when comparisons were conducted with the two fingers remaining separate, for all measures examined (threshold, slope and goodness of fit).

### Training task

Either the left or right middle finger was selected to be trained (half participants trained on the left/right, randomised over participants), i.e. the finger homologous to the trained finger was not subjected to training.

While incorporating the same tactile grating stimuli, we used a different task for training than was used in testing. This was done to encourage participants to learn tactile features of the stimuli, rather than task requirements (Harrar et al., 2014). The training task was also loosely an orientation discrimination task, but with a different task format. On each training trial, a single grating was presented twice to the trained finger (not once, as in testing; ~1 s between subsequent presentations). Participants were asked to report whether the two stimulus presentations were oriented in the same direction (e.g. down-down) or different directions (e.g. down-across), also 2AFC (see Figure 1). Four grating sizes were used for training. These sizes were based on performance in the baseline testing block. They were also selected to be in the dynamic accuracy range – determined by calculation of the participant’s perceptual ‘threshold’ in the baseline (defined below).

Each of the four gratings was presented for a set of 10 trials (order randomised), and then a short break was taken. This was repeated three times, resulting in 120 total trials (30 trials × 4 gratings). Auditory feedback on accuracy was provided by headphones trial-by-trial to maximise learning (i.e. ‘correct’/‘incorrect’).

### Psychophysical measures

**Figure 2.**
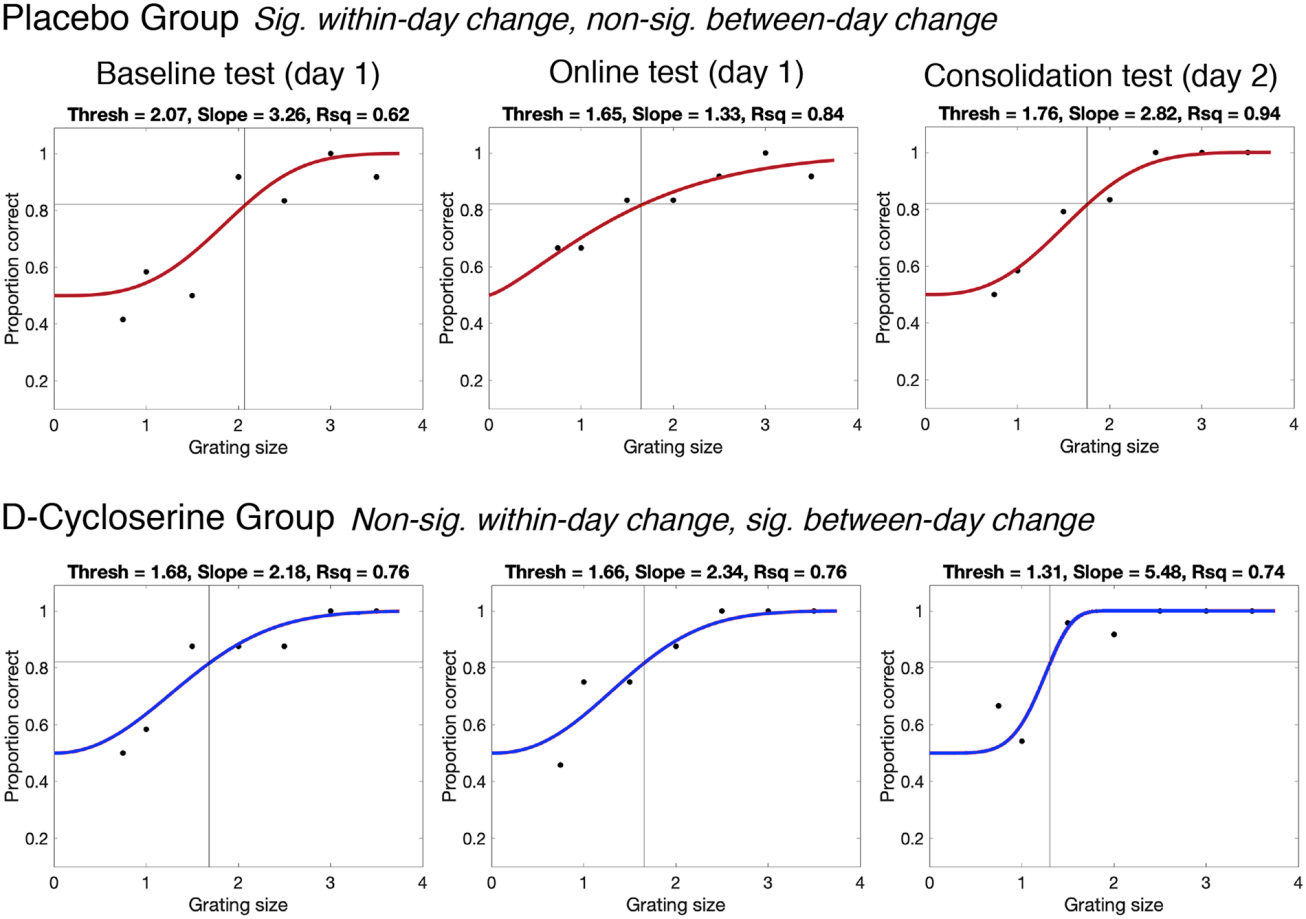
Psychometric functions for two representative participants from the placebo (red, top) and D-cycloserine (blue, bottom) groups from the three testing blocks – baseline, online and consolidation (from left to right). Tactile thresholds correspond with the grating (stimulus) value corresponding with the interpolated 82% accuracy value (represented by black crosshairs on the plot). The slope value is taken as the steepness of the psychometric function at the threshold point. Goodness of fit (*R*^2^) represents how well the psychometric function represented the data, i.e. how close the data points are to the line. See the placebo group at consolidation for a relatively shallow slope and lower *R*^2^, and the D-cycloserine group at consolidation for a steeper slope and improved fits (higher *R*^2^). ‘Sig.’ and ‘non-sig’ stand for significant and non-significant (*p* < 0.05), respectively.

As is typical in psychophysical studies of learning and perception, we examined the tactile ‘threshold’ as our primary measure of interest (Van Boven and Johnson, 1994; Vega-Bermudez and Johnson, 2001). This measure represents accuracy in the testing task across all difficulty levels of the testing task (grating sizes, see Figure 2). It was calculated by plotting accuracy as a function of grating size for a single testing block (separately for each finger). A psychometric curve was fitted to the data (using a non-linear regression with least squares fit). From this curve, we then interpolated the grating size corresponding with 82% accuracy (i.e. the threshold; see Supplementary Material for more details regarding choice of 82% accuracy level). Low threshold values indicate superior tactile perception.

As discussed in the Introduction, we extracted two other measures representing properties of the psychometric function – the ‘slope’ and ‘goodness of fit’. This was done to give a comprehensive account of task performance and provide other mechanistic information about how D-cycloserine might alter perceptual performance and learning (Gold et al., 2010; Wichmann and Hill, 2001). The slope value represents the steepness of the psychometric curve at the point where the threshold was interpolated (here, 82% accuracy). This measure, therefore, represents the difference in performance (accuracy) as a function of stimulus difficulty level (grating size). For instance, a high slope value is seen when there is a sharp difference in accuracy for easy stimuli and hard stimuli (see plots of different slope values in Figure 2). A low slope indicates little variation in performance as a function of task difficulty. This can occur, for example, due to cognitive lapses/inattention causing low accuracy on stimuli that should be well within the perceptual range (Klein, 2001; Swanson and Birch, 1992; Wichmann and Hill, 2001). Slope can also flatten with learning, as accuracy increases for ‘difficult’ stimuli (Gold et al., 2010).

Finally, we extracted goodness of fit (*R*^2^), which represent success in fitting the psychometric function to the data. This can be informative about noise in the data (Klein, 2001), as it represents the amount of variability in accuracy scores around the psychometric function, as a proportion of the total variation.

Across the entire dataset (both groups), *R*^2^ values were good, i.e. high values (M=0.71, standard error (SE)=0.02). As is typical, in some cases the psychometric functions did not fit the data well (*R*^2^<0.2). These cases were excluded from further analyses as the fits do not represent the data, and are thus, uninformative (cases excluded=13: D-cycloserine=3, Placebo=10; no differences between groups, see below). Removal of these cases improved *R*^2^ (M=0.75, SE=0.02).

### Psychometric function fitting failures

In a minimal number of cases (i.e. where a ‘case’ is data for a single participant/finger/block dataset), a psychometric function could not be fit to the data due to poor data quality. In 16 of 216 (total) cases, the curve was able to be regenerated following removal of a single outlying accuracy score (for one grating size). In the remaining four cases, a curve could not be fit because accuracy did not exceed 82% for any grating size; in these instances, this missing value was replaced with the maximum stimulus value (3.5 mm) (Dempsey-Jones et al., 2019).

### Experimental design and statistical analysis

A series of generalised estimating equation (GEE) analyses (Ballinger, 2004; Twisk, 2004) were used to analyse the data because the GEE is better able to directly account for the interdependence of the data (multiple fingers of the same participant, multiple testing blocks) compared with analysis of variance (ANOVA) methods. Please note, however, re-analysis with ANOVAs or linear mixed models produced the same picture of results overall (see Supplementary Material for further rationale regarding the use of GEE).

The experiment followed a mixed 3 × 2 × 2 design, with two within-participants factors, (testing) ‘Block’ (3 levels: baseline, online, consolidation), (tested) ‘Finger’ (two levels: trained, homologous), and one between-participants factor, ‘Group’ (2 levels: D-cycloserine, placebo). As discussed above, given non-significant differences between the two fingers tested (see Supplementary Material), these were collapsed for our analyses. This resulted in a 3 × 2 mixed design with factors Block and Group. The primary dependent variable of interest was tactile threshold. Analyses were then repeated for the slope and goodness of fit dependent variables.

The first, 3 × 2 analysis looked at all three testing blocks together. Following identification of an interaction, we then performed two 2 × 2 analyses to determine where learning occurred in the time course (baseline vs online, within-day learning; online vs consolidation, between-day learning). To supplement this we performed direct between-group comparisons, separately for each block (one-way GEE, with the factor Group; though note these between-group comparisons are not central to the results of interest which look at within-group changes to better index learning over time).

All previous analyses looked at performance in the testing blocks. As a final comparison, we looked at accuracy in the training task for the two groups. This allowed us to further probe for differences in tactile perception and performance while the drug was in its active phase.

All analyses were performed the IBM SPSS Statistics software package (Version 22.0; IBM Corp., Armonk, New York, USA).

## Results

### Thresholds vary differently over time as a function of drug

**Figure 3.**
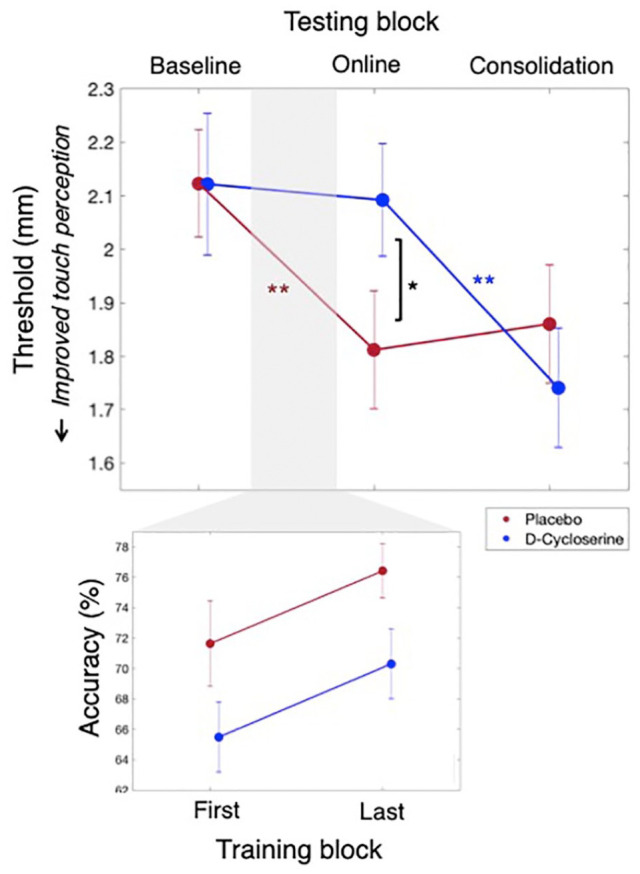
Tactile testing (top): Tactile perception thresholds averaged over the trained and homologous finger for the D-cycloserine (blue) and placebo (red) groups. A series of generalised estimating equation (GEE) analyses revealed significant within-day learning (baseline-online tests) for the placebo, but not D-cycloserine groups. In contrast, significant between-day learning was found for the D-cycloserine, but not placebo groups. This led to significant group differences at the online test (with worse thresholds for the D-cycloserine group), but not the baseline or consolidation tests. Tactile training accuracy (bottom): overall the D-cycloserine group had worse accuracy on the training task (main effect of Group), but both groups improved in performance over training blocks. For all comparisons, *indicates significance at *p* ⩽ 0.05, **indicates significance at *p* ⩽ 0.001.

We found administration of D-cycloserine before tactile training caused a difference in learning across the three testing blocks, as compared to the administration of a placebo control. This was revealed by a 3 × 2 mixed GEE with the within-participants factor Block (three levels: baseline, online, consolidation) and the between-participants factor Group (two levels: D-cycloserine, placebo), resulting in a significant interaction of Block × Group, χ^2^(2)=18.01, *p*<0.001; Table 2 column (a); Figure 3, top panel).

**Table 2.** Table of generalised estimating equation (GEE) results for tactile threshold data – within-participants comparisons (wp) are presented in the top rows, and between-group comparisons in lower rows (see Table 3 for slope and *R*^2^). Please note: all analyses were conducted using the averaged threshold of the trained and homologous finger (as these were non-significantly different, see Methods).

	(a)	(b)	(c)		(d)	(e)	
	Mixed GEE, 3 blocks (baseline, online, consolidation)	Mixed GEE, 2 blocks (baseline-online)	Follow-up wp GEE, 2 blocks (baseline-online)		Mixed GEE, 2 blocks (online-consolidation)	Follow-up wp GEE, 2 blocks (online-consolidation)	
			Cycloserine	Control		Cycloserine	Control
Comparison	Statistics (threshold)						
Block	χ^2^(2)=37.30, ***p*** <**0.001**^a^	χ^2^(1)=4.29, *p*=0.38	χ^2^(1)=0.12, *p*=0.729	χ^2^(1)=12.14, ***p*** =**0.001**^a^	χ^2^(1)=11.88, ***p*** =**0.001**^a^	χ^2^(1)=31.72, ***p*** <**0.001**^a^	χ^2^(1)=0.17, *p*=0.684
Group	χ^2^(1)=0.09, *p*=0.764	χ^2^(1)=0.66, *p*=0.415			χ^2^(1)=0.77, *p*=0.380		
Block × Group	χ^2^(2)=18.01, ***p*** <**0.001**^a^	χ^2^(1)=6.80, ***p*** =**0.009**^b^			χ^2^(1)=16.48, ***p*** <**0.001**^a^		
	(f)	(g)	(h)				
	Mixed GEE, baseline	Mixed GEE, online	Mixed GEE, consolidation				
Comparison							
Group	χ^2^(1)=0.30, *p*=0.587	χ^2^(1)=5.57, ***p*** =**0.018**^b^	χ^2^(1)=0.18, *p*=0.668				

Critical results related to the shifted time-course of learning are highlighted in green and grey.

a Indicates significance at *p*=0.001 and ^b^indicates significance at *p*=0.05.

Following this interaction up, we found that on the first day tactile thresholds changed differently over blocks as a function of drug administration. This was indicated by a 2 × 2 mixed GEE with the within-participants factor Block (two levels: baseline, online) and between-participants Group (two levels: D-cycloserine, placebo) with a significant Group × Block interaction, χ^2^(1)=6.80, *p*=0.009.

We then performed separate within-participant GEEs to determine whether the baseline vs online comparison was significant for the placebo group and D-cycloserine group alone. The placebo group showed improvement between tests on the first day, indicated by a significant main effect of Block (*p*<0.001, see Table 2 column (c)), where thresholds at the online test were significantly lower than at baseline (note: threshold drops indicate improvement in touch perception). In the D-cycloserine group, we saw no improvement between these tests. That is, the same analysis revealed no main effect of Block (*p*=0.729), indicating no significant change in thresholds between the baseline and online tests (see Table 2 column (c), Figure 3, top panel).

### Between-day learning: online vs consolidation tests

We then looked at learning gains occurring between days. This revealed – in direct contrast to the within-day learning results – that there was significant between-day learning in the D-cycloserine group, but not in the placebo group.

This was indicated by a 2 × 2 mixed GEE with the within-participants factor Block (two levels: online test, consolidation test) and between-participants Group (two levels: D-cycloserine, placebo) that produced a significant Group × Block interaction, χ^2^(1)=16.48, *p*<0.001. Follow-up within-participants GEEs revealed a significant improvement between tests for the D-cycloserine group, indicated by a main effect of Block (*p*<0.001, see Table 2 column (e)): where tactile thresholds improved significantly overnight between the online and consolidation tests. The placebo group, however, did not improve, showing no main effect of Block (*p*=0.684). Thus, there was no further change in thresholds for the placebo group following the significant drop the day before (see Table 2 column (e), Figure 3, top panel).

### Between-group comparisons

We next conducted direct between-group comparisons that indicated the administration of D-cycloserine prior to training changed the progression of learning, rather than the total amount of learning – which was the same between groups at the final testing point.

This was demonstrated by three one-way GEE analyses (one per block) with the factor Group. This revealed a non-significant group difference at baseline (*p*=0.587), but higher (worse) thresholds for the D-cycloserine group (M=2.09, SEM=0.10) than the placebo group (M=1.76, SEM=0.99) at the online test (*p*=0.018), and no significant difference at the consolidation test (*p*=0.668). Please see Table 2 column (f) to (h), for the first to last testing blocks, respectively. Also note that the between-groups difference at the online test drops just below significance if Bonferroni corrected for three comparisons (*p*=0.018, α_corr_=0.017). This may suggest that power could have been improved through a larger sample size though, as we note above, this result is non-critical, meant only to supplement the main within-group comparisons. All other results of interest remain with Bonferroni correction.

### Training accuracy worse for D-cycloserine group

Looking at performance accuracy of the tactile training task (as opposed to testing performance, analysed above), we found more evidence of interference in touch perception and performance while the drug was in its active phase. Specifically, the D-cycloserine group had significantly reduced accuracy on the training task as compared to the placebo group. This difference was stable over the testing blocks (see Figure 3, bottom panel).

This result was demonstrated by a mixed GEE with the within-participants factor Block (two levels: first training block, last training block) and the between-participants factor Group (two levels: D-cycloserine, placebo). This analysis produced a significant main effect of Block, χ^2^(1)=6.75, *p*=0.009, a significant main effect of Group, χ^2^(1)=5.56, *p*=0.018, and a non-significant interaction of Block × Group, χ^2^(1)=0.01, *p*=0.992. Descriptive statistics indicated that the D-cycloserine group had lower training task accuracy overall (averaged over blocks; M=68.27%, SEM=1.76) than the placebo group (averaged over blocks; M=73.16%, SEM=2.11).

### Slope and goodness of fit

**Figure 4.**
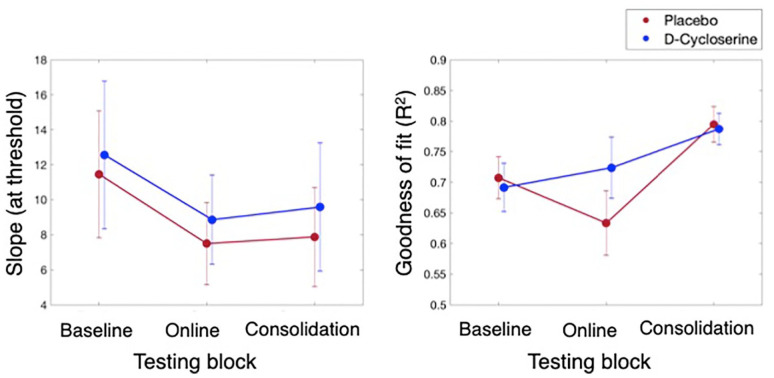
Slope (left) and goodness of fit (*R*^2^ right) results for the D-cycloserine (blue) and placebo (red) groups across the three testing blocks. No differences were found as a function of group for either measure. A main effect of Block was found for goodness of fit, whereby fitting success improved by the final testing block (averaged over groups; more details in text).

Our analyses of the slope data indicated there were no differences in perceptual sensitivity as a function of drug administration. This was shown by 3 × 2 GEE with the factors Block (baseline, online, consolidation) and Group (D-cycloserine, placebo) with the dependent variable slope. Indicating null effects of the drug, there was a non-significant main effect of Group (*p*=0.780), and non-significant interaction of Block × Group (*p*=0.960). Please see Table 3 column (a) and Figure 4, left panel for results. The main effect of Block was also non-significant (*p*=0.548), despite an apparent visual trend towards a decrease in slope values over blocks (Ballinger, 2004).

**Table 3.** Table of generalised estimating equation (GEE) results for slope data ((a) left) and goodness of fit/*R*^2^ ((b) right). Please note: all analyses were conducted with data collapsed for the trained and homologous fingers (as these were non-significantly different, see Methods).

	(a)	(b)
	Mixed GEE 3 blocks (baseline, online, consolidation)	Mixed GEE 3 blocks (baseline, online, consolidation)
Comparison	Statistics (slope)	Statistics (*R*^2^)
Block	χ^2^(2)=1.20, *p*=0.548	χ^2^(2)=16.77, ***p*** <**0.001**^a^
Group	χ^2^(1)=0.08, *p*=0.780	χ^2^(1)=0.55, *p*=0.457
Block × Group	χ^2^(2)=0.08, *p*=0.960	χ^2^(2)=2.90, *p*=0.235

a Indicates significance at *p*=0.001.

We also found that there were no significant differences in goodness of fit over block as a function of drug administration. This was demonstrated by a 3 × 2 GEE (factors as above) with goodness of fit (*R*^2^) as the dependent variable. As with slope, the group effects were non-significant: Group, *p*=0.457; Block × Group, *p*=0.235. Please see Table 3 column (b) and Figure 4, right panel for results. Also see the Supplementary Material for a Bayesian analysis to further support the non-significance of this interaction. The main effect of Block, however, was significant (*p*<0.001). Follow-up pairwise comparisons of the estimated marginal means indicated there was a significant increase in goodness of fit values at the final, consolidation test (M=0.80, SE=0.02) as compared to both the baseline (M=0.72, SE=0.02; *p*=0.001) and the online tests (M=0.69, SE=0.04; *p*=0.001). There was no difference between the baseline and online tests (*p*=0.289).

## Discussion

In this study we demonstrate that administration of D-cycloserine before tactile training modulated the process of tactile perceptual learning. Administration of D-cycloserine caused interference in within-day learning, where learning that occurred in the placebo group directly after training was not evident in the D-cycloserine group. Looking at learning between-days, the pattern reversed. The D-cycloserine group improved in tactile acuity, but the placebo group showed no learning additional to the within-day learning they demonstrated on the first day. Subsequently, because of greater relative gains overnight, the D-cycloserine group ‘caught up’ to the placebo group by the consolidation testing – reflected in non-significant differences in tactile thresholds at final testing.

While D-cycloserine has been shown to alter processes of memory, procedural learning and motor learning (see Introduction), it has not previously been shown that D-cycloserine can modulate perceptual (sensory) learning. Interestingly, Schwartz et al. (1996) report D-cycloserine improved performance on an implicit memory task in Alzheimer’s patients. However, because their task involved discriminating the same set of visually degraded words over 10 weeks, it could be that, in fact, the drug was improving patients’ ability to visually recognise and retain the degraded words. This may represent a form of perceptual learning, as well as/instead of memory function per se (also see Forsyth et al., 2015).

Here we show a clear modulation of the processes of tactile learning by D-cycloserine. While the drug does not confer a perceptual advantage at the final testing block for the D-cycloserine group, it may be that drug effects could continue to evolve over a longer period. Though this needs to be directly explored. Importantly, because of our longitudinal design we were able to capture the eventual ‘catch up’ in tactile thresholds for the D-cycloserine group. Failure to document this effect could have led to the erroneous conclusion that the drug impairs perceptual learning (rather than simply modulating its time-course). Regardless, our results have implications concerning at what time-point effects of D-cycloserine and similar drugs should be assessed, whether applied in a therapeutic or learning setting.

### A ‘delay’ in perceptual learning

Consistent with our predictions regarding interference, our results suggest D-cycloserine delayed learning that would normally occur concurrent to training until sufficient time had passed, possibly until the drug had been metabolised (half-life 8–15 h: Patel et al., 2011; van Berckel et al., 1997, 1998) or until sleep had occurred (see Feld et al., 2013). Given both groups show equivalent thresholds by the final testing block, this indicates that there was indeed learning in both the drug and placebo groups – where ‘learning’ is defined as significant improvement in perception from baseline testing (Johnson and Phillips, 1981; Van Boven and Johnson, 1994; Vega-Bermudez and Johnson, 2001). This means that training did have an effect on the D-cycloserine group, permitting eventual improvement in touch perception. This suggests that processing was initiated at the time of training, but may have only been completed or expressed later (e.g. during the consolidation period: Kuriyama et al., 2011).

While surprising, this picture is consistent with our findings from the training block. The D-cycloserine group was found to be impaired overall in training accuracy compared to the placebo group (main effect of group), suggesting some detrimental impact while the drug was active. However, the parallel learning curves of the two groups (no interaction of training block and group) indicate both groups improved at the training task equivalently over the duration.

Deeper analysis of other properties of the psychometric functions may provide some limited additional insight into the nature of this apparent delay or detriment. We show there was no difference in slope as a function of drug administration – overall or over time. There were also no differences in the goodness of fit of the psychometric functions to the data as a function of drug (see Bayesian supporting analysis in the Supplementary Material). These results suggest that while the D-cycloserine group failed to improve in touch thresholds directly after training on day 1, as was seen in the placebo group, there is no indication of gross attentional lapses (as can be seen in reduced slope, e.g. see Wichmann and Hill, 2001), or clear increases in noise in the data (as can be seen in large drops in function fitting success, also see Wichmann and Hill, 2001). Demonstrating the informational value of goodness of fit: our data showed the *R*^2^ values significantly improved (better fits) at the final testing block, compared to the previous two blocks. Thus, noise dropped with repeated testing and training (Klein, 2001). Given the non-significant nature of these results with respect to the drug, however, they will not be discussed further to avoid over interpretation of null results.

### Mechanism of action

Pharmacologically, D-cycloserine binds to the glycine site of NMDA receptors. Glycine is a co-agonist of these receptors, thus, they only open when both glycine and glutamate bind. This means that D-cycloserine increases the likelihood that glutamate release will activate NMDA receptors. D-cycloserine has an agonist effect on NMDA receptors when endogenous levels of glycine are low, i.e. by mimicking glycine’s effects (Lanthorn, 1994; Schneider et al., 2000). However, when levels of glycine are high, D-cycloserine has an antagonistic effect on NMDA receptors (Watson et al., 1990).

While D-cycloserine has been demonstrated to affect learning-related plasticity in the hippocampus and amgydala (LeDoux, 2000; Onur et al., 2010), it has also been shown to modulate prefrontal activity. For example, D-cycloserine has recently been shown to modulate human decision-making (Scholl et al., 2014) and modulate prefrontal activity in clinically phobic individuals viewing threatening stimuli (Aupperle et al., 2009). More generally, NMDA receptor activity has been linked with other higher-order cognitive functions such as working memory activity in the dorsolateral prefrontal cortex (Wang et al., 2013) and attention (Herrero et al., 2013). Looking at other NMDA-related compounds, D-serine – the primary endogenous co-agonist at the glycine site of synaptic NMDA receptors – has been suggested to improve auditory plasticity in a tone learning task after repeated administrations (Kantrowitz et al., 2016), and has been linked with performance on cognitive tasks (Panizzutti et al., 2019); also, see Guercio and Panizutti (2018) for other evidence of D-serine as a cognitive enhancer. Similarly, compounds that inhibit D-amino acid oxidase (DAO) may have the potential to improve cognition because DAO degrades D-amino acids such as D-serine, though initial results are mixed (Pritchett et al., 2016), also see (Mahmoud et al., 2019). Indeed, when DAO inhibitors such as sodium benzoate have been used to treat schizophrenia (Lane et al., 2013; Tsai et al., 1998) and early-stage Alzheimer’s disease (Lin et al., 2014) they have been found to produce improvements in neurocognitive functioning, as an adjunct to improvements in symptoms of clinical pathology. Of particular relevance, Lane et al. (2013) showed DOA inhibitor administration produced improvements in schizophrenic patients on a visual learning task.

While some suggest perceptual learning occurs in lower-order, primary sensory area neurons (Adab and Vogels, 2011; Jehee et al., 2012; Schoups et al., 2001; Shibata et al., 2011), a growing body of evidence suggests that such learning critically depends on changes in prefrontal or decision-making areas (Kahnt et al., 2011; Law and Gold, 2008; Petrov et al., 2005; Zhang et al., 2010). This is thought to occur as a function of improved read-out of neurons in sensory brain regions by higher-order areas (Gold et al., 2010). It may be, therefore, that D-cycloserine acts to alter perceptual learning by modulating the signal-to-noise ratio in these higher-order brain areas: sharpening or blunting perception of the tactile stimuli by affecting neural tuning (Law and Gold, 2008).

While most studies report a positive or null effect of NMDA agonist drugs on learning and cognition (see Introduction), high doses of D-cycloserine have caused impairment visual discrimination performance similar in magnitude to impairments caused by an NMDA-receptor antagonist (Schneider et al., 2000). Our previous work also indicates a worsening of reaction times in a motor task as a function of NMDA receptor agonist activity (Gunthner et al., 2016; see Supplementary Material regarding sensorimotor interaction effects: Sanders et al., 2019). It may be important to consider that different neurocognitive mechanisms could have differing optimal dose ranges. Consequently, for some tasks a ‘typical’ dose may be more likely to increase the risk over-potentiation, and thus, NMDA agonist-like effects (Lanthorn, 1994). This may also depend on divergent baseline NDMA levels in different neural regions/circuits, known to modulate NMDA-related effects (Standage and Pare, 2011). It is likely, however, only through electrophysiological work with non-humans that we may know the precise mechanistic action of NMDA partial-agonists on perceptual learning. Consistently, the range of doses between different studies (ranging from 50–500 mg for single session learning studies; see Introduction) could also influence physiological response and thus should be considered in understanding learning and cognition outcomes.

## Conclusion

We demonstrate that D-cycloserine administration prior to tactile training altered the typical process of perceptual learning, seen in controls. We suggest that the findings of this study could provide a new and simple explanation for some of the variation in previous research studies of the effect of NMDA agonists on learning and cognition. Specifically, it may be important to consider learning-related drug effects that vary over time or for different learning processes. Expounding these mechanisms will, in all likelihood, permit greater consistency in the application of this potentially useful resource to the optimisation of learning and memory.

## Supplemental Material

sj-pdf-1-jop-10.1177_0269881120986349 – Supplemental material for Human perceptual learning is delayed by the N-methyl-D-aspartate receptor partial agonist D-cycloserine Supplemental material, sj-pdf-1-jop-10.1177_0269881120986349 for Human perceptual learning is delayed by the N-methyl-D-aspartate receptor partial agonist D-cycloserine by Harriet Dempsey-Jones, Susann Steudte-Schmiedgen, Michael Browning, Tamar R Makin, Marcella L Woud, Catherine J Harmer, Juergen Margraf and Andrea Reinecke in Journal of Psychopharmacology
