# Supplementary material for: Human perceptual learning is delayed by the N-methyl-D-aspartate receptor partial agonist D-cycloserine
Source: J Psychopharmacol. 2021 Feb 11;35(3):253–64. doi: 10.1177/0269881120986349 (PMC7924109; doi:10.1177/0269881120986349)
Supplement: sj-pdf-1-jop-10.1177_0269881120986349 – Supplemental material for Human perceptual learning is delayed by the N-methyl-D-aspartate receptor partial agonist D-cycloserine [file sj-pdf-1-jop-10.1177_0269881120986349.pdf]

Supplementary Materials

Extended Methods

Full exclusion criteria and screening

Exclusion criteria were: history of DSM-IV axis-I diagnosis, neurological or serious medical illness such as epilepsy or renal insufficiency, first-degree family member with a history of a severe psychiatric disease, CNS-active medication during the last 6 weeks, current medication with cycloserine, ethionamide or isoniazid, pregnancy or breast-feeding, smoking, a body mass index (BMI) outside the range 18-30 kg/m2, and insufficient English skills. Participants were assessed using the Structured Clinical Interview for DSM-IV (SCID; First et al. 1997) to establish the absence of DSM-IV axis I psychiatric disorders.

General procedure

Upon arrival on day 1, participants underwent a short familiarisation with the tactile stimuli before completing the baseline test. In the familiarisation test participants were presented with a small number of presentations of the largest grating stimulus (3.5mm, i.e., the 'easiest' stimulus, in terms of how difficult it was to perceptually discriminate the orientation of the ridges (/grooves) by touch). Participants were presented with this stimulus 6 times, 3 times in the 'down' and 3 in the 'across' orientation (see main text for orientation definitions). In the familiarisation, the presentations of the stimulus were accompanied with verbal labels (i.e., experimenter presented the grating in the down orientation, and spoke 'down' aloud). This was done to help participants match the percept to the labels required to complete the task, resolving some ambiguity in how to perform the task, and improving the reliability of the initial testing trials. After familiarisation, on day 1, participants completed a baseline tactile test, then were administered with either the drug or placebo. Following this they performed the tactile training, and finally, completed the online tactile test (more details below).

The final day (24/ 48 hours later, see main text) involved the final (consolidation) tactile test only. Following this, a demand check was completed. The demand check asked participants whether they believed they were in the placebo or control group (‘yes’ or ‘no’). Participants were also asked to respond regarding any side effects they believed they experienced (as an open-ended question).

Grating selection & presentation

As discussed in the main text, the testing task incorporated 7 grating sizes (1 grating per block; 12 trials per block). Each of the seven gratings was presented for one block, and then 4 more grating sets were represented for one block each – based on the participant’s task accuracy. The computer started with the four largest (i.e., easiest) gratings as the set selected for representation. If accuracy had been on/ above 90% on any of these gratings, they were not re-presented. This meant the set of gratings used for the final block were selected as a function of participant performance – allowing a range to be presented that were within the dynamic accuracy range (i.e., the area of the psychometric function were response to stimuli was variable – not consistently accurate).

For the training task, grating difficulties were selected that sat around the participant’s individual perceptual ‘threshold’ determined from performance in the baseline test. The perceptual threshold is the grating size (i.e., stimulus difficulty level) corresponding with a specific level of perceptual/ task accuracy. While this specific accuracy level chosen for the perceptual threshold is technically an arbitrary criterion, there are standard values that are consistently used in the field. These accuracy levels usually either correspond with the point at which a participant is at chance accuracy (0/50%), or a level were participants begin to show reliably high accuracy that is not yet at ceiling, e.g., either 75% or 82% accuracy (Sathian and Zangaladze, 1996; Sathian and Zangaladze, 1997; Van Boven and Johnson, 1994). Here, we used the 82% accuracy point to determine the perpetual threshold. This point is used as it corresponds to 1 standard deviation above

the upper bound of perceptual certainty – also arbitrarily, though consistently defined in the literature as 75% accuracy (Smith et al., 1997).

Two gratings were selected for training that sit above the threshold level and two below, for instance, threshold = 1.89mm, training gratings = 1.2, 1.5, 2.0, and 2.5mm. Each of the 4 training gratings was presented once (order randomised), followed by a short break (~1-2 minutes, participants were encouraged to remove their blindfold and take a rest). This was repeated three times, resulting in 120 training trials in total.

Generalised Estimating Equation Analysis

GEE analysis was selected for use in the current study because this method allows a high degree of control regarding explicit specification of the working correlation matrix (i.e., within-subjects dependencies) between dependent variables, thus, providing a better fitting model (Ballinger, 2004). Here, the working correlation matrix was set to exchangeable, which indicates the structure has homogenous correlations between elements, i.e., compound symmetry structure (Field, 2009). Please note, the picture of results remained largely consistent with analysed with alternate methods (i.e., linear mixed models, ANOVA).

Extended Results and Discussion

No difference in tactile thresholds between fingers

Initial analyses revealed there were no differences in the way tactile thresholds changed over block/ group as a function of finger tested. This was revealed by a mixed 3 x 2 x 2 GEE with factors Block (baseline, online, consolidation), Finger (trained, homologous), and Group (D-cycloserine, placebo). This analysis revealed no main effects or interactions with the factor Finger (.196 $< p >$ .523. Please see Table S1. This suggested that the thresholds for the two fingers be collapsed to simplify further analyses – see main text.

Table S1. *GEE analysis of thresholds assessing consistency in change over blocks/ groups as a function of finger.*

	**A**<br>Mixed GEE, All tests<br>(baseline, online, consolidation)
**Comparison**	**Statistics**
Block	$\chi^2(2) = 39.27$, **$p < .001$****
Finger	$\chi^2(1) = 0.59$, $p = .441$
Group	$\chi^2(1) = 0.12$, $p = .726$
Block x Finger	$\chi^2(2) = 1.05$, $p = .593$
Block x Group	$\chi^2(2) = 17.76$, **$p < .001$****
Finger x Group	$\chi^2(1) = 0.60$, $p = .438$
Block x Finger x Group	$\chi^2(2) = 3.26$, $p =.196$

Bayesian follow-up of goodness of fit effects

As discussed in the main text, when using a 3 x 2 GEE with factors Block (baseline, online, consolidation) and Group (D-cycloserine, placebo) to investigate slope results we found that there was a non-significant interaction of Block x Group ($p = .235$). However, visual inspection revealed an apparent divergence in the R^2 values at the second test (online test). To probe whether this difference at the online test was indeed non-valid as the interaction suggested, we performed a Bayesian Independent Samples comparison (using the Bayesian toolbox in SPSS Version 25). The default prior was used, and we tested for BF_{01} (null/ alternate hypothesis). This analysis returned a Bayes Factor of 3.84, meaning there was moderate evidence in support of the null hypothesis – that both the D-cycloserine and placebo groups had the same mean goodness of fit value at the online test. This supported the lack of difference indicated by the interaction.

Motor cortical activity and tactile perception

There is a developing understanding in the somatomotor research community that the somatosensory and motor cortices share a tight, two-way connection (Darainy et al., 2013; Tame et al., 2015; Omrani et al., 2014; Sanders et al., 2019). Indeed, the representation of the hand in primary somatosensory cortex (SI), particularly in Brodmann Area 3b, has recently been shown to be driven by patterns of daily hand action, i.e., motor synergies (Ejaz, Hamada, & Diedrichsen, 2015; Ingram, Körding, Howard, & Wolpert, 2008). Motor action of the fingers also causes efferent signals into SI from the motor system (Wolpert & Flanagan, 2001; Wolpert, Ghahramani, & Jordan, 1995). Similarly, some evidence suggests that motor cortical cells respond directly to somatosensory stimuli, e.g., proprioceptive positioning (Fromm et al., 1984). Given our previous work suggesting that D-cycloserine can have a negative impact on reaction times during a motor learning task (Gunthner et al., 2016) it may be important to consider that the temporary interference in tactile learning we document here could be, in some part, related to changes in the motor system. Other research also suggests D-cycloserine can influence motor cortical function. Particularly, Nitsche et al. (2004) looked at motor cortex excitability, as reflected in motor evoked potentials following transcranial magnetic stimulation. While they found that D-cycloserine did not alter motor cortical excitability on its own, it did cause an increase the duration – but not magnitude – of *tDSC-induced* motor cortex excitability. Direct research is needed to investigate whether sensory-motor interactions could impact tactile processing, as well as the direction of any identified effects (i.e., is the motor cortex driving somatosensory changes, or vice versa). Similarly, changes in both areas may be related to changes in the tuning of higher-order, read-out areas, particularly decision making areas (see main text). Studies that look at networks level changes across the whole brain will likely be critical in understanding the causality of D-cycloserine effects.

Tactile perception changes following training versus repetitive stimulation

Here, we show improvements in tactile perception for the trained finger, as well as in the homologous finger of the other hand. This is consistent with

previous studies showing perceptual improvements spread to fingers that share a topographic relationship with the trained finger, e.g., the adjacent and homolgous fingers (Dempsey-Jones et al., 2016; Dempsey-Jones et al., 2019; Harrar et al., 2014), or in a topographic gradient (Harris and Diamond, 2000; Harris et al., 1999). Also see Tamè et al. (2011) who showed a tactile mask does not affect perception when it is on the homologous finger to that being stimulated (in a go-no-go tactile decision-making task).

Other studies of tactile plasticity following repetitive somatosensory stimulation (RSS) protocols show a different pattern regarding where tactile improvements are seen. For instance, RSS studies show perceptual improvements on the trained finger, and other fingers that *do not* share a topographic relation with the trained finger, i.e., no change for the homologous finger (no other fingers tested; Muret et al., 2014), or no change in the homologous and adjacent fingers, but learning in all others tested (thumb, index, and middle tested; Macchione et al., 2018).

While tactile learning studies show gains in topographically related fingers, and RSS studies show inhibition of learning in these same fingers – both types of somatosensory plasticity must depend at least in part on receptive field properties in primary somatosensory cortex. Specifically, they likely depend largely on neuronal architecture in Brodmann Area 3b, which is known to contain neurons representing the trained and adjacent fingers, as well as neurons representing the trained and homologous fingers (Detorakis and Rougier, 2014; Thakur et al., 2012). Thus, while apparently in conflict regarding improvement or lack-thereof, both paradigms show consistency in perceptual outcome for the untrained adjacent and homologous fingers – within studies.

This leads to the question of whether tactile learning and RSS-related changes are supported by different mechanisms (though both in some way dependent on the same topographic organisation). It may be that RSS is better conceptualised not as ‘learning’ per se (or the result of ‘training’ effects), because participants do not pay any attention to, and are often

unaware of, the tactile stimulation properties (with many protocols allowing participants to continue tasks of daily life during stimulation, e.g., Muret et al., 2014).

Further, creating a distinction between tactile learning and RSS may be warranted because perceptual changes following RSS do not follow some typical principles of ‘learning’ common across learning studies across modalities. For example, RSS effects disappear the next day (Dinse et al., 2006; Dinse et al., 2003), while tactile learning consolidates overnight – as with other forms of learning (Dempsey-Jones et al., 2016; Dempsey-Jones et al., 2019).

What neural changes might play into these two mechanisms? RSS is thought to cause an increase in the size of the stimulated finger in somatosensory cortex as a result of Hebbian co-stimulation principles (with fMRI: Hodzic et al., 2004; indexed with SSEP; Dinse et al., 2006). Such changes, indeed, could support the sensory improvements seen for the trained finger.

Regarding the topographic inhibition documented in more recent RSS studies (e.g., Macchione et al., 2018), the pattern of spread must be related to receptive field properties (homologous and adjacent are inhibited, but not other fingers) – the mechanism by which this occurs is unclear. Unfortunately, limited mechanistic description is available in the RSS literature, and the precise details will likely only be elucidated by electrophysiology in non-humans – which is currently unavailable for RSS studies, but has been explored in tactile learning in rodents (Harris et al., 1999; Harris et al., 2001).

References

Ballinger G. (2004) Using Generalised Estimating Equations for Longitudinal Data Analysis. *Organisational Research Methods* 7: 127-150.

Darainy M, Vahdat S and Ostry DJ. (2013) Perceptual learning in sensorimotor adaptation. *Journal of Neurophysiology* 110: 2152-2162.

Dempsey-Jones H, Harrar V, Oliver J, et al. (2016) Transfer of tactile perceptual learning to untrained neighbouring fingers reflects natural use relationships. *J Neurophysiol*: jn 00181 02015.

Dempsey-Jones H, Themistocleus AC, Carone D, et al. (2019) Blocking tactile input to one finger using anaesthetic enhances touch perception and learning in other fingers. *Journal of Experimental Psychology: General*.

Detorakis GI and Rougier NP. (2014) Structure of receptive fields in a computational model of area 3b of primary sensory cortex. *Frontiers in computational neuroscience* 8.

Dinse HR, Kleibel N, Kalisch T, et al. (2006) Tactile coactivation resets ageational model of area 3b of primary sensory cortex*Annals of neurology* 60: 88-94.

Dinse HR, Ragert P, Pleger B, et al. (2003) Pharmacological modulation of perceptual learning and associated cortical reorganization. *Science* 301: 91-94.

Field A. (2009) Discovering statistics using SPSS. *Sage Publishing, Thousand Oaks, CA* 3rd Edition.

Fromm C, Wise SP and Evans EV. (1984) Sensory response properties of pyramidal tract neurons in the precentral motor cortex and postcentral gyrus of the rhesus monkey. *Experimental Brain Research* 54: 177-185.

Gunthner J, Scholl J, Favaron E, et al. (2016) The NMDA receptor partial agonist d-cycloserine does not enhance motor learning. *J Psychopharmacol* 30: 994-999.

Harrar V, Spence C and Makin TR. (2014) Topographic generalization of tactile perceptual learning. *Journal of experimental psychology. Human perception and performance* 40: 15-23.

Harris JA and Diamond ME. (2000) Ipsilateral and contralateral transfer of tactile learning. *Neuroreport* 11: 263-266.

Harris JA, Harris IM and Diamond ME. (2001) The topography of tactile learning in humans. *The Journal of Neuroscience* 21: 1056-1061.

Harris JA, Petersen RS and Diamond ME. (1999) Distribution of tactile learning and its neural basis. *Proc Natl Acad Sci U S A* 96: 7587-7591.

Hodzic A, Veit R, Karim AA, et al. (2004) Improvement and Decline in Tactile Discrimination Behavior after Cortical Plasticity Induced by Passive Tactile Coactivation. *24* 4.

Macchione S, Muret D, Koun E, et al. (2018) RSS-induced tactile improvement transfers from one hand to the other one. *Hand, Brain & Technology Conference*.

Muret D, Dinse HR, Macchione S, et al. (2014) Touch improvement at the hand transfers to the face. *Current Biology* 24: R736-R737.

Nitsche MA, Jaussi W, Liebetanz D, et al. (2004) Consolidation of Human Motor Cortical Neuroplasticity by D-Cycloserine. *Neuropsychopharmacology* 29.

Omrani M, Murnaghan CD and Pruszynski JA. (2014) Distributed task-specific processing of somatosensory feedback for voluntary motor control. *Elife*.

Sanders ZB, Wesselink D, Dempsey-Jones H, et al. (2019) Similar somatotopy for active and passive digit representation in primary somatosensory cortex. *Biorxiv*: 754648.

Sathian K and Zangaladze A. (1996) Tactile spatial acuity at the human fingertip and lip - Bilateral symmetry and interdigit variability. *Neurology* 46: 1464-1464.

Sathian K and Zangaladze A. (1997) Tactile learning is task specific but transfers between fingers. *Perception & Psychophysics* 59: 119-128.

Smith JD, Shields WE, Schull J, et al. (1997) The uncertain response in humans and animals. *Cognition* 62: 75-97.

Tame L, Farne A and Pavani F. (2011) Spatial coding of touch at the fingers: Insights from double simultaneous stimulation within and between hands. *Neuroscience letters* 487: 78-82.

Tame L, Pavani F, Braun C, et al. (2015) Somatotopy and temporal dynamics of sensorimotor interactions: evidence from double afferent inhibition. *Eur J Neurosci* 41: 1459-1465.

Thakur PH, Fitzgerald PJ and Hsiao SS. (2012) Second-order receptive fields reveal multidigit interactions in area 3b of the macaque monkey. *Journal of Neurophysiology* 108: 243-262.

Van Boven RW and Johnson KO. (1994) The limit of tactile spatial resolution in humans Grating orientation discrimination at the lip, tongue, and finger. *Neurology* 44: 2361-2361.
